# Acute Kidney Injury Associated With Remdesivir: A Comprehensive Pharmacovigilance Analysis of COVID-19 Reports in FAERS

**DOI:** 10.3389/fphar.2022.692828

**Published:** 2022-03-25

**Authors:** Bin Wu, Min Luo, Fengbo Wu, Zhiyao He, Yuwen Li, Ting Xu

**Affiliations:** ^1^ Department of Pharmacy, West China Hospital, Sichuan University, Chengdu, China; ^2^ West China School of Pharmacy, Sichuan University, Chengdu, China

**Keywords:** remdesivir, COVID-19, acute kidney injury, pharmacovigilance analysis, FAERS

## Abstract

Acute kidney injury (AKI) is a common complication among patients with the novel coronavirus (COVID-19). COVID-19 along with AKI usually resulted in a poor prognosis for those affected. Remdesivir is a novel antiviral drug that was urgently approved for the treatment of COVID-19. In the current study, safety data of remdesivir were limited. We gathered information on COVID-19 cases in patients with adverse events that were reported to the U.S. Food and Drug Administration (US FDA) Adverse Event Reporting System (FAERS) database. We employed the reporting odds ratio (ROR) method to perform disproportionality analysis. Finally, we identified 12,869 COVID-19 cases. A total of 3,991 of these cases reported remdesivir as a primary suspected drug, while 8,878 cases were treated with other drugs. More AKI events occurred in cases of male patients and those above the age of 65 years. We detected a significant association between remdesivir and AKI: ROR = 2.81, 95% CI (2.48, 3.18). The association was stronger after the propensity score matching ROR = 3.85, 95% CI (3.11, 4.78). The mean time to AKI event onset was 4.91 ± 7.25 days in COVID-19 cases with remdesivir therapy. The fatality proportion was 36.45% in AKI cases with remdesivir treatment. This pharmacovigilance study identified a significant association between AKI events and remdesivir treatment in COVID-19 patients by mining FAERS real-world big data. Although causality was not confirmed, the association between remdesivir and AKI should not be ignored, especially in the older, male COVID-19 inpatients.

## Introduction

We are suffering from a global pandemic of COVID-19, which is caused by severe acute respiratory syndrome coronavirus 2 (SARS-CoV-2). This pandemic is a serious threat to both public health and economic stability around the world.

Nearly 80% of COVID-19 patients were reported to experience mild to moderate symptoms, including fever, cough, or fatigue (Wu and McGoogan, 2020). However, some severe complications and even death occurred in older or high-risk patients (Wu and McGoogan, 2020). AKI is reported to be a complication in patients with severe COVID-19 (Chan et al., 2021). The reported incidence of AKI in COVID-19 patients varied from 0.5 to 46% (Guan et al., 2020; Hirsch et al., 2020; Yang et al., 2020; Chan et al., 2021; Diebold et al., 2021). The pathophysiology of AKI in COVID-19 patients is not known; however, potential mechanisms of developing AKI include direct SARS-CoV-2 infection in the kidney, immune response dysregulation, or as a result of multi-organ failure (Huang et al., 2020; Su et al., 2020). Other evidence has indicated that AKI in COVID-19 patients might be associated with renal toxic treatment (Zheng et al., 2020).

Remdesivir, a novel antiviral drug, was approved by the U.S. Food and Drug Administration (US FDA) for the treatment of hospitalized COVID-19 patients. Remdesivir could shorten hospital stays and reduce mortality in COVID-19 patients (Bansal et al., 2020). The remdesivir formulation contains sulfobutylether-β-cyclodextrin (SBE-*β*-CD) (Adamsick et al., 2020; NIH, 2021), which is excreted through the kidney and has some renal toxicity. For this reason, clinical studies excluded renal insufficiency recipients with an estimated glomerular filtration rate (eGFR) of <30 ml/min (Beigel et al., 2020; Wang et al., 2020) or eGFR <50 ml/min (Joyner et al., 2020; Goldman et al., 2021). The renal toxicity of remdesivir is not yet fully understood, so renal function should be monitored in patients undergoing remdesivir treatment. One study tried to analyze adverse events of remdesivir in COVID-19 patients with or without severe renal impairment (SRI, creatinine clearance <30 ml/min) and discovered that a higher proportion of patients experienced serum creatinine elevations in the SRI group (Pettit et al., 2020). Other analyses, using the WHO safety database (Chouchana et al., 2021; Gérard et al., 2021), detected significant association between nephrotoxicity and remdesivir. However, the safety data of remdesivir on renal function were limited.

Adverse event reporting system data were an outstanding source for post-marketing drug safety monitoring and pharmacovigilance study. The US FDA Adverse Event Reporting System (FAERS) is one of the largest databases open to the public (FDA, 2018). The objective of the present study was to detect the association between remdesivir treatment and the potential risk of AKI in COVID-19 cases by systematically assessing spontaneous reports that were submitted to the FAERS database.

## Article Types

### Study Design and Data Sources

This was a retrospective study carried out to analyze the AKI events related to remdesivir treatment in COVID-19 cases that were reported in the FAERS pharmacovigilance databases. The FAERS data were extracted from the FAERS Quarterly Data Extract Files, available at https://fis.fda.gov/extensions/FPD-QDE-FAERS/FPD-QDE-FAERS.html. This study analyzed data between January 2004 and December 2020. We extracted cases with confirmed COVID-19 from the FAERS database and divided these cases into the remdesivir group, which were treated with remdesivir as a primary suspected drug, and the control group, which were treated with other primary suspected drugs. We then compared the AKI events between groups by disproportionality analysis. Data on COVID-19 cases were collected, including case ID, indication, suspected drug, adverse event, serious outcome, occurrence country, reporter type, sex, age, treatment date, and event date.

### Materials and Methods

The FAERS database consisted of seven data tables: The “DEMO” table for patient demographic information, the “DRUG” table for drug information, the “REAC” table for adverse event information, the “OUTC” table for patient outcome information, the “RPSR” table for report source information, the “THER” table for drug therapy date information, and the “INDI” table for drug indication. We managed FAERS data by Microsoft SQL server 2017 software.

We first deduplicated reported cases, following the FDA recommendation. We removed the same records from the “DEMO” table and left one and then deleted the earliest “FDA_DT” column when the “CASEID” column was the same. We also removed the lower “PRIMARYID” column when the “CASEID” and “FDA_DT” columns were the same.

We identified cases with COVID-19 indication in the “INDI” table, according to the Standardized Medical Dictionary for Regulatory Activities Queries (SMQs) version 23.1 (ICH, 2021). The SMQ narrow search for COVID-19 cases consisted of 18 preferred terms (PT) (Supplementary Table S1). We then identified AKI events in the “REAC” table using SMQ narrow search (19 PTs, Supplementary Table S2). For cases that reported more than one PT of the same SMQ, we removed duplicate records and retained one. For example, if one case reported two records of “coronavirus infection” and “coronavirus test positive,” we counted the two records as one COVID-19 case.

We then identified cases that were treated with remdesivir in both the “drugname” and “prod_ai” columns using “remdesivir” and “VEKLURY” in the “DRUG” table. This restricted the “role_cod” as the primary suspected drug.

We further estimated the time from infection to the onset of the AKI event. We unified all dates, which were then formatted as yyyy-mm-dd. The time to the event was calculated using the event date (EVENT_DT) in the “DEMO” table minus the drug start date (START_DT) in the “THER” table. In order to ensure the accuracy of this calculation, we excluded cases without full year, month, and day data. We also excluded cases that had the event date listed earlier than the drug start date.

Finally, we analyzed serious outcomes including death, life-threatening conditions, hospitalization, disability, congenital anomaly, required intervention to prevent permanent impairment or damage, and other serious events. If a case reported more than one outcome, we kept the more serious one. For example, one case reported both death and hospitalization, and we kept the outcome of death.

In order to verify the robustness of the results, we also performed sensitivity analysis in three independent methods. First, we reidentified AKI cases using SMQ broad search (52 PTs, Supplementary Table S2) instead of narrow search. Second, we reidentified remdesivir with both primary and secondary suspected drugs. Third, we chose the top five primary suspected drugs as the new control group, including hydroxychloroquine, azithromycin, bamlanivimab, tocilizumab, and lopinavir\ritonavir.

#### Statistical Analysis

We compared the risk of AKI associated with remdesivir-treated cases against AKI reported from other drugs in COVID-19 cases from the FAERS database.

We used the reporting odds ratio (ROR) method for disproportionality analysis. The ROR compared the potentially increased risk of AKI events for remdesivir with the same adverse events for other primary suspected drugs (Supplementary Table S3). We then performed propensity score matching (PSM) analysis to balance variables between groups. The 1:1 PSM analysis was conducted by using the PSM model of SPSS 25.0 software and included variables available from FAERS such as patient age, sex, reporter type, and the country of occurrence. These were sampled without replacement, and we used optimal matching of both exact match and fuzzy match, with a matching tolerance of 0.001 (Yao et al., 2017; Johnson et al., 2018). The missing data were treated as an independent classification. For example, the sex variable was classified as female, male, and unknown for missing data. We then calculated an adjusted ROR based on matching data. The ROR signal criterion was defined as the lower limit of ROR 95% CI exceeding one (van Puijenbroek et al., 2002).

We compared the variables between remdesivir and control groups using Pearson’s chi-squared test both before and after PSM. We compared each serious outcome between AKI and non-AKI cases of remdesivir or the control group using Pearson’s chi-squared test or Fisher’s exact probability method. A *p* value of less than 0.05 indicated a significant difference. These statistical analyses were conducted using SPSS version 25.0 (IBM corporation, Armonk, New York, United States).

## Results

### COVID-19 Case Identification in the FDA Adverse Event Reporting System

**FIGURE 1:**
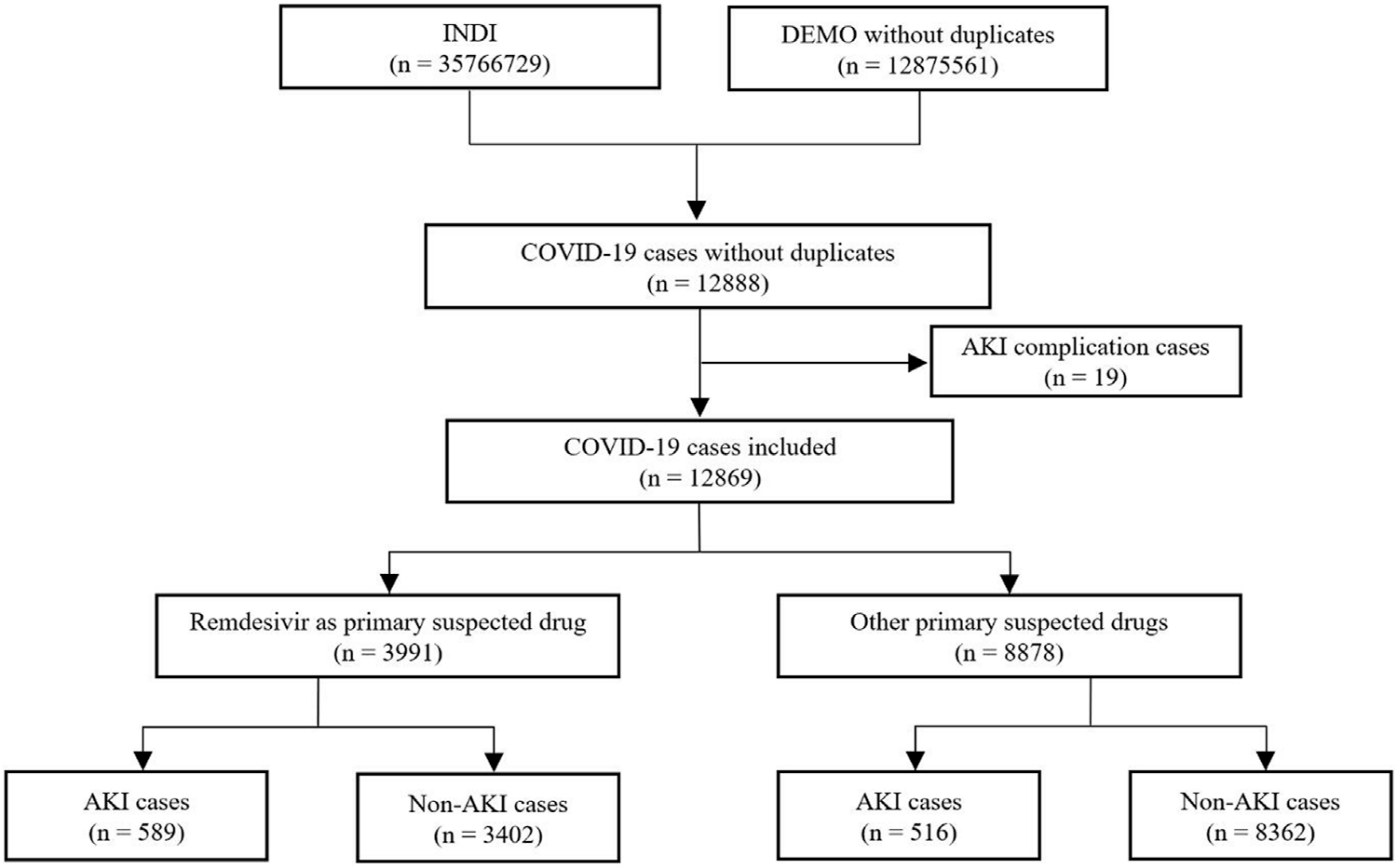
Flowchart of identifying AKI cases in COVID-19 patients from the FAERS database.

We identified a total of 12,888 adverse event cases with an indication of COVID-19 from the FAERS database. Of these, 19 cases with a complication of AKI were excluded. Finally, we included 12,869 COVID-19 cases in disproportionality analysis, 3,991 cases in the remdesivir group and 8,878 cases in the control group, with 589 and 516 AKI cases in each group, respectively. The details of case identification are shown in Figure 1.

### Characteristics of COVID-19 Cases Reported in the FDA Adverse Event Reporting System

The characteristics of the 12,869 COVID-19 cases are shown in Table 1. Both groups indicated a higher proportion in the ≥65 age group, especially those AKI cases in the remdesivir group (56.88%). The male-to-female ratio was 2.55 in the control-AKI group, followed by 1.95 in the remdesivir-AKI group. More than 80% of cases were reported by health professionals, including physicians, pharmacists, and other healthcare professionals. More than 90% of remdesivir adverse event cases occurred in the United States. All cases were reported in the year 2020, except one that was reported in June 2019.

**TABLE 1:** Characteristics of COVID-19 cases reported in the FAERS database.

Characteristic	Remdesivir				Control				Total/N
	AKI		Non-AKI		AKI		Non-AKI		
	*n*	%	*n*	%	*n*	%	*n*	%	
Age (years)									
<18	6	1.02	49	1.44	2	0.39	164	1.96	221
18–44	38	6.45	574	16.87	67	12.98	1,112	13.30	1791
45–64	200	33.96	1,069	31.42	128	24.81	2,586	30.93	3,983
≥65	335	56.88	1,565	46.00	223	43.22	2,976	35.59	5,099
Unknown	10	1.70	145	4.26	96	18.60	1,524	18.23	1775
Sex									
Female	198	33.62	1,335	39.24	120	23.26	2,650	31.69	4,303
Male	386	65.53	2024	59.49	306	59.30	4,471	53.47	7,187
Unknown	5	0.85	43	1.26	90	17.44	1,241	14.84	1,379
Type of reporter									
Health professional	571	96.94	3,257	95.74	427	82.75	7,076	84.62	11,331
Non-health professional	4	0.68	47	1.38	29	5.62	734	8.78	814
Unknown	14	2.38	98	2.88	60	11.63	552	6.60	724
Occurrence country									
United States	551	93.55	3,198	94.00	194	37.60	3,137	37.51	7,080
Spain	2	0.34	8	0.24	77	14.92	1,309	15.65	1,396
France	4	0.68	26	0.76	105	20.35	899	10.75	1,034
Italy	0	0.00	10	0.29	32	6.20	939	11.23	981
China	0	0.00	0	0.00	7	1.36	235	2.81	242
Japan	7	1.19	29	0.85	7	1.36	163	1.95	206
Brazil	4	0.68	12	0.35	4	0.78	121	1.45	141
Turkey	0	0.00	0	0.00	0	0.00	131	1.57	131
Portugal	8	1.36	34	1.00	16	3.10	59	0.71	117
United Kingdom	2	0.34	9	0.26	8	1.55	95	1.14	114
Other countries	11	1.87	76	2.23	66	12.79	1,274	15.24	1,427

After 1:1 PSM by patient age, sex, reporter type, and occurrence country, and with a matching tolerance of 0.001, a total of 4,642 cases were gathered with 2,321 cases in each group. The differences of age, sex, reporter type, and country of occurrence between the remdesivir and the control group were not significant. The characteristics of COVID-19 cases after PSM are shown in Table 2.

**TABLE 2:** Characteristics of COVID-19 cases reported in the FAERS database after propensity score matching.

Characteristic	Remdesivir		Control		Pearson chi-square value	*p* Value
	*n*	%	*n*	%		
COVID-19 cases	2,321	100.00	2,321	100.00	—	—
Age (years)						
<18	48	2.07	54	2.33	1.06	0.90
18–44	340	14.65	357	15.38		
45–64	735	31.67	715	30.81		
≥65	1,046	45.07	1,045	45.02		
Unknown	152	6.55	150	6.46		
Sex						
Female	974	41.96	970	41.79	0.80	0.67
Male	1,300	56.01	1,295	55.79		
Unknown	47	2.02	56	2.41		
Type of reporter						
Health professional	2,159	93.02	2,151	92.68	0.24	0.89
Non-health professional	50	2.15	54	2.33		
Unknown	112	4.83	116	5.00		
Occurrence country						
United States	2,118	91.25	2,124	91.51	32.39	0.22
Portugal	35	1.51	36	1.55		
Japan	36	1.55	31	1.34		
France	30	1.29	25	1.08		
Brazil	16	0.69	34	1.46		
Germany	13	0.56	10	0.43		
Italy	10	0.43	10	0.43		
Poland	7	0.30	13	0.56		
Spain	10	0.43	3	0.13		
United Kingdom	11	0.47	2	0.09		
Other countries	35	1.51	33	1.42		

### Disproportionality Analysis

The disproportionality analysis indicated a significant association between AKI events and remdesivir treatment in COVID-19 patients. The risk of AKI events reported with remdesivir was 2.81 times greater than those reported with the other primary suspected drugs. These details are shown in Supplementary Table S4. After PSM, the risk ratio was even higher, ROR = 3.85, 95% CI (3.11, 4.78) (Table 3).

**TABLE 3:** Comparison of acute kidney injury events in COVID-19 cases reported in the FAERS database.

Population	AKI	Remdesivir/*n*	Control/*n*	ROR	95% CI for ROR	Pearson chi-square value	*p* Value
Before PSM	Yes	589	516	2.81	(2.48, 3.18)	280.73	<0.000
	No	3,402	8,362				
After PSM	Yes	394	117	3.85	(3.11, 4.78)	168.73	<0.000
	No	1927	2,204				

The sensitivity analysis indicated robust outcome (Supplementary Table S5).

### Time to Event Onset

**FIGURE 2:**
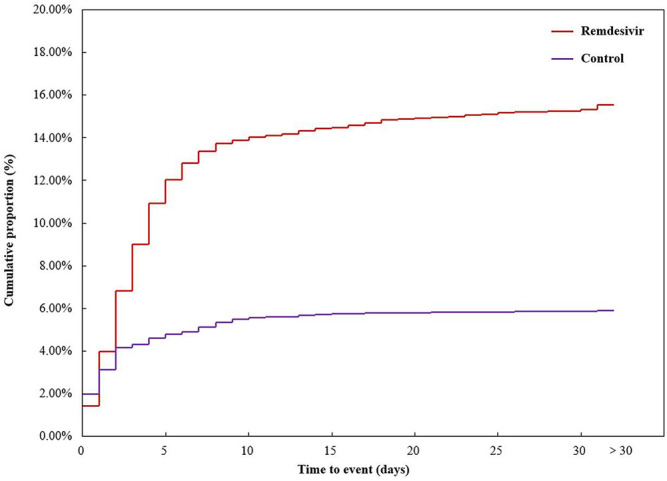
Cumulative proportion of time to acute kidney injury event onset. Outcomes of COVID-19 cases.

A total of 6,790 COVID-19 cases were reported with sufficient data and included in time analysis. There were 3,361 cases in the remdesivir group (522 AKI cases) and 3,429 cases in the control group (202 AKI cases). The mean time to AKI event onset was 4.91 ± 7.25 days in the remdesivir group and 3.03 ± 4.97 days in the control group, *p* = 0.001. The cumulative proportion of time to the AKI event onset is shown in Figure 2.

A total of 11,128 COVID-19 cases reported outcome data. The proportion of death and life-threatening outcomes was significantly higher in AKI cases than that in non-AKI cases in both the remdesivir and control groups. Each serious outcome of COVID-19 cases reported in the FAERS database is shown in Table 4.

**TABLE 4:** Outcomes of COVID-19 cases reported in the FAERS database.

Outcome	Remdesivir (*N* = 3,027)				*p* Value	Control (*N* = 8,101)				*p* Value
	AKI		Non-AKI			AKI		Non-AKI		
	*n*	%	*n*	%		*N*	%	*n*	%	
Total	524	100.00	2,503	100.00	—	494	100.00	7,607	100.00	—
Death	191	36.45	794	31.72	0.0357	155	31.38	1,567	20.6	<0.000
Life-threatening	38	7.25	117	4.67	0.0149	63	12.75	532	6.99	<0.000
Hospitalization	84	16.03	317	12.66	0.0388	144	29.15	2,124	27.92	0.5558
Disability	6	1.15	8	0.32	0.0225^a^	0	0.00	30	0.39	0.2579^a^
Congenital anomaly	0	0.00	2	0.08	1.0000^a^	0	0.00	2	0.03	1.0000^a^
Required intervention	9	1.72	34	1.36	0.5415^a^	1	0.20	4	0.05	0.2700^a^
Other serious events	196	37.40	1,231	49.18	<0.000	131	26.52	3,348	44.01	<0.000

a Examined by using Fisher’s exact probability method. Others were examined by using Pearson’s chi-squared test.

## Discussion

The COVID-19 pandemic challenged scientists, physicians, and other health professionals to urgently find treatment methods for patients suffering from COVID-19. During this endeavor, remdesivir, a SARS-CoV-2 nucleotide analog RNA polymerase inhibitor, was approved for the treatment of COVID-19 patients requiring hospitalization. Clinical experience and evidence of this new antivirus drug were limited, especially due to lack of remdesivir safety data. We performed the first pharmacovigilance study to establish the association between AKI adverse events and remdesivir treatment using the FAERS database. Our study provided new evidence for the potential risk of was not a rare complication in hospitalized patients with COVID-19, especially in critical patients (Chan et al., 2021). The prognosis of COVID-19 patients with AKI complication was poor (Hirsch et al., 2020). In order to reduce the risk of bias affected by the COVID-19 disease, our study only included patients with COVID-19 disease and excluded those with AKI before medication in order to compare the risk of AKI in remdesivir treatment to that in other treatments. The results of disproportionality analysis indicated a significant association between remdesivir use and AKI adverse events. After PSM by patient age, sex, reporter type, and occurrence country, the association between remdesivir and AKI event was found to be stronger. A pharmacovigilance analysis, based on the World Health Organization (WHO) VigiBase database, gathered 138 AKI cases (SMQ broad search) associated with remdesivir treatment among COVID-19 patients and discovered a ROR of AKI with remdesivir was 20.30, 95% CI (15.70, 26.30), compared with the control group (hydroxychloroquine, tocilizumab, and lopinavir\ritonavir) (Gérard et al., 2021). Another study, also based on the WHO VigiBase database, gathered data on 5,532 COVID-19 cases and revealed a higher risk of AKI events in patients treated with remdesivir than those treated with chloroquine, hydroxychloroquine, dexamethasone, sarilumab, or tocilizumab, ROR = 7.2, 95% CI (5.7, 9.0) (Chouchana et al., 2021). A randomized, controlled trial compared both 5 and 10 days of remdesivir treatment for severe COVID-19 patients. Results found that four AKI cases occurred in the group that had 5 days of treatment, as compared to 15 AKI cases in the group that had 10 days of treatment. (Goldman et al., 2021). Additional clinical studies discovered adverse event cases of kidney injuries as well (Grein et al., 2020; Wang et al., 2020). The high disproportionality of AKI events in COVID-19 patients with remdesivir treatment is not yet fully understood. We speculated that COVID-19 induced a high morbidity of renal injury (Armaly et al., 2021; Sharma et al., 2021) when COVID-19 patients with renal injury (eGFR < 30 ml/min) were given remdesivir. Of note, this treatment was not recommended to patients with an eGFR of less than 30 ml/min. Kidney functions of COVID-19 patients may be further damaged by remdesivir. As a result, the risk of AKI in COVID-19 patients undergoing remdesivir therapy was increased. Although causality was not confirmed, the association between remdesivir treatment and AKI risk should be further assessed.

When analyzing the age of patients in the selected COVID-19 cases, we found the proportion of AKI adverse events was higher in patients above 65 years of age. The VigiBase pharmacovigilance study revealed that the median age of remdesivir-related kidney disorder cases was 65 years, with an interquartile range of 55–73 years (Chouchana et al., 2021). Since older patients were more likely to suffer severe COVID-19 and experience worse outcomes (Lim et al., 2020; Shahid et al., 2020), more attention should be paid to the older population when they are receiving remdesivir treatment.

Gender was another bias for the severity and mortality of COVID-19 (Pradhan and Olsson, 2020; Alwani et al., 2021). Our study discovered that the male-to-female ratio was 1.67 in all included COVID-19 adverse event cases. The sex disparities should be multifactorial due to the differences in comorbidity, lifestyle (Alwani et al., 2021), and immune system function (Pradhan and Olsson, 2020) between male and female patients.

Our study gathered 522 AKI cases with remdesivir treatment for time-to-event analysis. More than half (303, 58.05%) of these cases had an AKI adverse event occur within 3 days, and 404 (77.40%) cases had an AKI event occur within 5 days. Remdesivir was recommended to hospitalized COVID-19 patients for a period of 5 days, for those patients without invasive mechanical ventilation and/or ECMO, and for 10 days, for those patients without clinical improvement by day 5 of remdesivir treatment or those patients requiring invasive mechanical ventilation and/or ECMO (Administration, 2020). Renal function should be carefully monitored in COVID-19 patients who received remdesivir treatment.

More than one-third of the COVID-19 cases with AKI events reported in the FAERS eventually passed away. The death proportion was calculated by taking the number of patients that died from COVID-19 with AKI adverse events reported in the FAERS and dividing this by all COVID-19 cases with adverse events reported in the FAERS. The death proportion was higher in the remdesivir group than that in the control group and was found to be 2.30–16.31% higher than mortality reported in all COVID-19 patients (Kang and Jung, 2020; Matta et al., 2020). There are a few things that may explain the higher death proportion. First, remdesivir was given only to COVID-19 inpatients who were suffering from more severe diseases and had a worse prognosis than outpatients. Second, COVID-19 patients with AKI events were in worse conditions overall than those patients without renal damage. Therefore, COVID-19 inpatients with AKI events should be evaluated more carefully.

The current retrospective study had several limitations. FAERS, a spontaneous reporting system, is voluntary and open to the public, so under- or over-reporting, along with missing information, was inevitable (Palleria et al., 2013). Some calculations, especially time-to-event analysis, only included cases with sufficient data reported. Although PSM was performed, we could not find and balance all risk factors between groups based on the FAERS data, which provided limited patient information. Although our study revealed a significant association between AKI and remdesivir treatment in COVID-19 patients, causality between this adverse event and use of this drug could not be determined based on the FAERS data alone.

## Conclusion

This pharmacovigilance study identified a significant association between AKI events and remdesivir treatment in COVID-19 patients based on FAERS real-world data. Although causality was not confirmed, the association between remdesivir and AKI should not be ignored, especially in older, male COVID-19 inpatients. Renal function should be carefully monitored in COVID-19 patients being treated with remdesivir.

## Data Availability

The raw data supporting the conclusion of this article will be made available by the authors, without undue reservation.
