# Supplementary material for: Acute Kidney Injury Associated With Remdesivir: A Comprehensive Pharmacovigilance Analysis of COVID-19 Reports in FAERS
Source: Front Pharmacol. 2022 Mar 25;13:692828. doi: 10.3389/fphar.2022.692828 (PMC8990823; doi:10.3389/fphar.2022.692828)
Supplement: Supplementary file 1 [file Table1.docx]

Supplementary Material

# Supplementary Table

## Supplementary Table S1. SMQ narrow search for COVID-19. (SMQ code 20000237)

| **No.** | **Preferred term code** | **Preferred terms** |
| --- | --- | --- |
| 1 | 10051905 | Coronavirus infection |
| 2 | 10070255 | Coronavirus test positive |
| 3 | 10084268 | COVID-19 |
| 4 | 10084271 | SARS-CoV-2 test positive |
| 5 | 10084380 | COVID-19 pneumonia |
| 6 | 10084394 | Occupational exposure to SARS-CoV-2 |
| 7 | 10084451 | Suspected COVID-19 |
| 8 | 10084456 | Exposure to SARS-CoV-2 |
| 9 | 10084457 | COVID-19 immunisation |
| 10 | 10084458 | COVID-19 prophylaxis |
| 11 | 10084459 | Asymptomatic COVID-19 |
| 12 | 10084460 | COVID-19 treatment |
| 13 | 10084461 | SARS-CoV-2 carrier |
| 14 | 10084480 | SARS-CoV-2 test false negative |
| 15 | 10084491 | SARS-CoV-2 antibody test positive |
| 16 | 10084639 | SARS-CoV-2 sepsis |
| 17 | 10084640 | SARS-CoV-2 viraemia |
| 18 | 10084767 | Multisystem inflammatory syndrome in children |

## Supplementary Table S2. SMQ narrow and broad search for AKI. (SMQ code 20000003)

Term scope 2: SMQ narrow search. Term scope 1 plus 2: broad search.

| **No.** | **Preferred term code** | **Preferred terms** | **Term scope** |
| --- | --- | --- | --- |
| 1 | 10002847 | Anuria | 2 |
| 2 | 10003885 | Azotaemia | 2 |
| 3 | 10018875 | Haemodialysis | 2 |
| 4 | 10029155 | Nephropathy toxic | 2 |
| 5 | 10030302 | Oliguria | 2 |
| 6 | 10034660 | Peritoneal dialysis | 2 |
| 7 | 10038435 | Renal failure | 2 |
| 8 | 10038447 | Renal failure neonatal | 2 |
| 9 | 10049776 | Renal impairment neonatal | 2 |
| 10 | 10049778 | Neonatal anuria | 2 |
| 11 | 10053090 | Haemofiltration | 2 |
| 12 | 10061105 | Dialysis | 2 |
| 13 | 10062237 | Renal impairment | 2 |
| 14 | 10066338 | Continuous haemodiafiltration | 2 |
| 15 | 10069339 | Acute kidney injury | 2 |
| 16 | 10069688 | Acute phosphate nephropathy | 2 |
| 17 | 10072370 | Prerenal failure | 2 |
| 18 | 10078987 | Foetal renal impairment | 2 |
| 19 | 10081980 | Subacute kidney injury | 2 |
| 20 | 10001580 | Albuminuria | 1 |
| 21 | 10005481 | Blood creatinine abnormal | 1 |
| 22 | 10005483 | Blood creatinine increased | 1 |
| 23 | 10005846 | Blood urea abnormal | 1 |
| 24 | 10005851 | Blood urea increased | 1 |
| 25 | 10011372 | Creatinine renal clearance decreased | 1 |
| 26 | 10018356 | Glomerular filtration rate abnormal | 1 |
| 27 | 10018358 | Glomerular filtration rate decreased | 1 |
| 28 | 10029117 | Nephritis | 1 |
| 29 | 10037032 | Proteinuria | 1 |
| 30 | 10038533 | Renal transplant | 1 |
| 31 | 10038537 | Renal tubular disorder | 1 |
| 32 | 10038540 | Renal tubular necrosis | 1 |
| 33 | 10046358 | Urea renal clearance decreased | 1 |
| 34 | 10048302 | Tubulointerstitial nephritis | 1 |
| 35 | 10049630 | Oedema due to renal disease | 1 |
| 36 | 10050335 | Renal tubular dysfunction | 1 |
| 37 | 10050760 | Blood urea nitrogen/creatinine ratio increased | 1 |
| 38 | 10053123 | Protein urine present | 1 |
| 39 | 10055003 | Creatinine urine decreased | 1 |
| 40 | 10059895 | Urine output decreased | 1 |
| 41 | 10061480 | Renal function test abnormal | 1 |
| 42 | 10062747 | Hypercreatininaemia | 1 |
| 43 | 10065673 | Nephritic syndrome | 1 |
| 44 | 10068447 | Creatinine renal clearance abnormal | 1 |
| 45 | 10069022 | Kidney injury molecule-1 | 1 |
| 46 | 10071021 | Creatinine urine abnormal | 1 |
| 47 | 10071503 | Crystal nephropathy | 1 |
| 48 | 10074739 | Intradialytic parenteral nutrition | 1 |
| 49 | 10075142 | Fractional excretion of sodium | 1 |
| 50 | 10077515 | Hyponatriuria | 1 |
| 51 | 10078933 | Renal tubular injury | 1 |
| 52 | 10082703 | Neutrophil gelatinase-associated lipocalin increased | 1 |

## Supplementary Table S3. Two-by-two contingency table for reporting odds ratio analysis.

AKI: acute kidney injury. ROR: reporting odds ratio. 95%CI: 95% confidence interval.

| **Primary suspected drugs** | **COVID-19 cases with AKI events** | **COVID-19 cases with all other adverse event** |
| --- | --- | --- |
| Remdesivir | a | b |
| All other drugs | c | d |
| ROR = $\frac{a/b}{c/d}$, 95%CI for ROR = exp (ln (ROR) ± 1.96$\sqrt{\frac{1}{a}+\frac{1}{b}+\frac{1}{c}+\frac{1}{d}}$) | | |

## Supplementary Table S4. Top 20 primary suspected drugs for COVID-19 cases reported in FAERS

ATC: Anatomic Therapeutic Chemical.

| **No.** | **ATC classification** | **ATC code** | **Drug name** | **Case number /N** | **Case proportion /%** |
| --- | --- | --- | --- | --- | --- |
| 1 | - | - | remdesivir | 3991 | 31.01 |
| 2 | P01 | P01BA02 | hydroxychloroquine | 1916 | 14.89 |
| 3 | J01 | J01FA10 | azithromycin | 1349 | 10.48 |
| 4 | - | - | bamlanivimab | 933 | 7.25 |
| 5 | L04 | L04AC07 | tocilizumab | 798 | 6.20 |
| 6 | J05 | J05AR10 | lopinavir\ritonavir | 600 | 4.66 |
| 7 | H02 | H02AB04 | methylprednisolone | 183 | 1.42 |
| 8 | L04 | L04AC14 | sarilumab | 156 | 1.21 |
| 9 | J05 | J05AH02 | oseltamivir | 146 | 1.13 |
| 10 | - | - | alcohol (hand sanitizer) | 138 | 1.07 |
| 11 | H02 | H02AB02 | dexamethasone | 111 | 0.86 |
| 12 | J01 | J01MA12 | levofloxacin | 85 | 0.66 |
| 13 | J01 | J01DD04 | ceftriaxone | 84 | 0.65 |
| 14 | L04 | L04AD02 | tacrolimus | 81 | 0.63 |
| 15 | L04 | L04AC08 | canakinumab | 69 | 0.54 |
| 16 | B01 | B01AB05 | enoxaparin | 66 | 0.51 |
| 17 | L04 | L04AC03 | anakinra | 62 | 0.48 |
| 18 | - | - | casirivimab\imdevimab | 62 | 0.48 |
| 19 | L04 | L04AA06 | mycophenolic acid | 55 | 0.43 |
| 20 | N02 | N02BE01 | paracetamol | 54 | 0.42 |

## Supplementary Table S5. Sensitivity analysis of acute kidney injury events in COVID-19 cases between groups reported in FAERS database

AKI: acute kidney injury. ROR: reporting odds ratio. 95%CI: 95% confidence interval. PS: primary suspected. SS: secondary suspected.

| **Sensitivity analysis method** | **AKI** | **Remdesivir** /n | **Control** /n | **ROR** | **95%CI for ROR** | **Pearson chi-square value** | ***P* value** |
| --- | --- | --- | --- | --- | --- | --- | --- |
| **AKI SMQ broad search** | | | | | | | |
|  | Yes | 837 | 575 | 3.83 | (3.42, 4.29) | 592.25 | ＜0.000 |
|  | No | 3154 | 8303 |  |  |  |  |
| **Remdesivir as PS and SS drug** | | | | | | | |
|  | Yes | 617 | 488 | 2.90 | (2.56, 3.29) | 298.54 | ＜0.000 |
|  | No | 3572 | 8192 |  |  |  |  |
| **Specific drugs as comparator** | | | | | | | |
| Hydroxychloroquine | Yes | 589 | 103 | 3.05 | (2.45, 3.79) | 110.18 | ＜0.000 |
|  | No | 3402 | 1813 |  |  |  |  |
| Azithromycin | Yes | 589 | 53 | 4.23 | (3.17, 5.65) | 111.79 | ＜0.000 |
|  | No | 3402 | 1296 |  |  |  |  |
| Bamlanivimab | Yes | 589 | 21 | 7.52 | (4.84, 11.69) | 108.99 | ＜0.000 |
|  | No | 3402 | 912 |  |  |  |  |
| Tocilizumab | Yes | 589 | 33 | 4.01 | (2.80, 5.75) | 66.41 | ＜0.000 |
|  | No | 3402 | 765 |  |  |  |  |
| Lopinavir\Ritonavir | Yes | 589 | 77 | 1.18 | (0.91, 1.52) | 1.56 | 0.212 |
|  | No | 3402 | 523 |  |  |  |  |
